# PCR-Based Genotyping of Zebrafish Genetic Mutants

**DOI:** 10.21769/BioProtoc.5248

**Published:** 2025-03-20

**Authors:** Swathy Babu, Yuko Nishiwaki, Ichiro Masai

**Affiliations:** Developmental neurobiology unit, Okinawa Institute of Science and Technology Graduate University, Onna, Japan

**Keywords:** Zebrafish, Genotyping, PCR, Mutant analysis, Developmental biology, Embryos, Restriction enzyme digestion, Phenotypic defects, Model organism

## Abstract

Zebrafish genetic mutants have emerged as a valuable model system for studying various aspects of disease and developmental biology. Mutant zebrafish embryos are generally identified based on phenotypic defects at later developmental stages, making it difficult to investigate underlying molecular mechanisms at earlier stages. This protocol presents a PCR-based genotyping method that enables the identification of wild-type, heterozygous, and homozygous zebrafish genetic mutants at any developmental stage, even when they are phenotypically indistinguishable. The approach involves the amplification of specific genomic regions using carefully designed primers, followed by gel electrophoresis. This genotyping method facilitates the investigation of the molecular mechanisms driving phenotypic defects that are observed at later timepoints. This protocol allows researchers to perform analyses such as immunofluorescence, RT-PCR, RNA sequencing, and other molecular experiments on early developmental stages of mutants. The availability of this protocol expands the utility of zebrafish genetic mutants for elucidating the molecular underpinnings of various biological processes throughout development.

Key features

• Enables genotyping of zebrafish genetic mutants at any developmental stage, even before the onset of phenotypic defects.

• Utilizes PCR amplification and restriction enzyme digestion to distinguish wild-type, mutant, and heterozygous genotypes.

## Background

Zebrafish (*Danio rerio*) have emerged as a powerful vertebrate model organism for studying various aspects of biology and development [1,2]. Zebrafish are easy to genetically manipulate and retain optical transparency during early developmental stages. They share 70% human gene orthologs [3], providing a great model to investigate molecular mechanisms underlying different biological processes and diseases. However, a common challenge arises when genetic mutations lead to phenotypic defects that only become visible at later developmental stages. In such cases, it can be difficult to study the underlying molecular mechanisms at earlier timepoints, as the wild-type and mutant embryos may appear phenotypically indistinguishable.

This protocol describes a genotyping method that enables the identification of wild-type, mutant, and heterozygous zebrafish embryos at any developmental stage, even before the onset of the mutant phenotype. The PCR-based genotyping approach utilizes carefully designed primers that amplify a region harboring the mutation of interest and allows for subsequent identification of genotypes through restriction enzyme digestion, regardless of whether the mutation creates or eliminates a restriction site.

By facilitating the investigation of molecular mechanisms at earlier developmental stages, this genotyping method expands the utility of zebrafish genetic mutants for elucidating the underlying biology of various developmental processes and diseases. It allows us to perform molecular analyses, such as immunofluorescence, RT-PCR, and RNA sequencing, on early developmental stages of mutants, enabling the understanding of the molecular events driving later-onset phenotypic defects. The protocol was validated using a specific zebrafish mutant, *banp^rw337^*, which exhibits a small eye phenotype at 4 days post-fertilization (dpf) due to cell death [4] and is embryonically lethal by 7–8 dpf. Before 4 dpf, the mutant embryos are phenotypically indistinguishable from their wild-type and heterozygous siblings. Overall, this protocol provides a valuable tool for the zebrafish research community, enabling the investigation of molecular mechanisms underlying genetic mutations at early developmental stages, even when the phenotypic differences are not apparent. This protocol has the potential to be adapted for other zebrafish mutants, further expanding the utility of this model organism in determining the molecular mechanisms underlying development and disease.

## Materials and reagents

**Biological materials**

1. Zebrafish genetic mutants

**Reagents**

1. N-Phenylthiourea (PTU) (Nacalai Tesque, catalog number: 27429-22)

2. Ethyl m-Aminobenzoate methanesulfonate (tricaine) (Nacalai Tesque, catalog number: 14805-82)

3. Methylene blue solution (Japan Pet Design Co., LTD., catalog number: 4975677045615)

4. Nonidet P-40 (NP40) (Nacalai Tesque, catalog number: 23640-94)

5. Polyoxyethylene sorbitan monolaurate (Tween 20) (Nacalai Tesque, catalog number: 28353-85)

6. Proteinase K (Nacalai Tesque, catalog number: 29442-14)

7. NuSieve3:1 agarose (LONZA, catalog number: 50090)

8. MboII enzyme (Takara Bio Inc, catalog number: 1145A); includes 10× (L) buffer

9. 1 kb Plus DNA ladder (NEB, catalog number: N3200S)

10. Paraformaldehyde, powder (PFA) (Nacalai Tesque, catalog number: 26126-54)

11. Phosphate buffer saline (PBS) tablets (Medicago, catalog number: 09-2051-100)

12. Ethanol (99.5%) (FUJIFILM Wako Pure Chemical Co, catalog number: 057-00451)

13. JB-4 Embedding kit (contains JB-4 solution A and B) (Polysciences, Inc., catalog number: 00226)

14. Toluidine Blue O (WALDECK, catalog number: 1B-481)

15. AmpliTaq Gold^TM^ DNA polymerase (Applied Biosystems, catalog number: 4486226)

16. 10× PCR gold buffer (Applied Biosystems, catalog number: 4486222)

17. 25 mM MgCl_2_ solution (Applied Biosystems, catalog number: 4486224)

18. 10 mM dNTP mix (Applied Biosystems, catalog number: 362275)

19. Inclusion reagent (Entellan^TM^ new Sigma-Aldrich, catalog number: 1.0790.0100)

20. NaCl (Nacalai Tesque, catalog number: 31333-45)

21. KCl (Nacalai Tesque, catalog number: 28514-75)

22. CaCl_2_·2H_2_O (Nacalai Tesque, catalog number: 06731-05)

23. MgSO_4_·7H_2_O (Nacalai Tesque, catalog number: 21003-75)

**Solutions**

1. E3 medium (see Recipes)

2. Embryo lysis buffer (see Recipes)

3. PCR master mix (see Recipes)

4. 1× PBS (see Recipes)

5. 10× PTU stock solution (see Recipes)

**Recipes**

**1. E3 medium**

**Table:** 

Reagent	Final concentration	Quantity or Volume
NaCl	5 mM	0.2922 g
KCl	0.17 mM	0.0126465 g
CaCl_2_·2H_2_O	0.33 mM	48.51 mg
MgSO_4_·7H_2_O	0.33 mM	81.3351 mg
Methylene blue solution	0.002%	200 μL
Deionized water		Up to 1 L

**2. Embryo lysis buffer (10 mL)**

**Table:** 

Reagent	Volume
10× PCR gold buffer	1 mL
NP40 (0.3% in total volume)	30 μL
Tween 20 (0.3% in total volume)	30 μL
MilliQ H_2_0	Up to 10 mL

**3. PCR master mix**

**Table:** 

Reagent	Quantity or Volume
10× PCR gold buffer	2.5 mL
10 mM dNTP mix (2.5 mM each)	0.5 mL
25 mM MgCl_2_ solution	1.5 mL
Deionized water	17.38 mL

*Note: PCR master mix can be prepared, aliquoted, and stored at -20 °C for up to one year.*

**4. 1**× **PBS**

**Table:** 

Reagent	Final concentration
NaCl	140 mM
KCl	2.7 mM
Phosphate buffer pH 7.4	10 mM

*Note: To prepare 1× PBS, measure 100 mL of deionized water at room temperature (approximately 25 °C) and add one PBS tablet (pH 7.4). Stir the solution thoroughly until the tablet is fully dissolved, ensuring no undissolved particles remain. Avoid using water warmer than 25 °C to maintain buffer stability.*

**5. 10× PTU stock solution (0.03%)**

**Table:** 

Reagent	Quantity or Volume
PTU	0.03 g
Deionized water	Up to 100 mL

**Laboratory supplies**

1. Metal mesh tea strainer (for egg collection) [Hibiki-an or any available brand: 2.95 inch (7.5 cm) diameter, 6.49 inch (16.5 cm) length]

2. Nets to catch zebrafish [NISSO, catalog number: RQ-16 (S size), 17 (M size)]

3. Petri dish (10 cm dish) (IWAKI, catalog number: SH90-15E)

4. Pasteur pipette (IWAKI, catalog number: IK-PAS-5P)

5. Transfer pipette (Samco Scientific, catalog number: 336)

6. Transparent flat embedding mold, Beem (Polysciences, Inc., catalog number: 23257)

7. Wooden block (2 cm) (AsOne, catalog number: 2-173-03)

8. Plastic sheets or cellophane paper

9. Glass slide [Matsunami Glass Ind Ltd., catalog number: S9901 (MAS coat), S2215]

10. Coverslip (No. 1), 24 × 60 mm (Matsunami Glass Ind Ltd., catalog number: C024601)

## Equipment

1. Zebrafish breeding tanks (Aquatic Habitats, model: 3L, Library)

2. Zebrafish mating tank (AQUA, model: crossing tank)

3. PCR machine, iCycler, thermal cycler (Bio-Rad, model: 170-8720)

4. Submarine electrophoresis apparatus (Bio-Craft, model: for 25 cm gel)

5. Large gel plate for 192 samples (Bio-Craft, model: 25 cm gel)

6. Microtome Rotatif (MICROM, model: HM335E)

7. Forceps (tip style: flat/non-toothed)

8. Hot plate, paraffin stretcher (Sakura Finetek Japan Co., model: PS-52)

9. Microscope (Zeiss, model: AxioPlan2)

## Software and datasets

**Figure 1.**
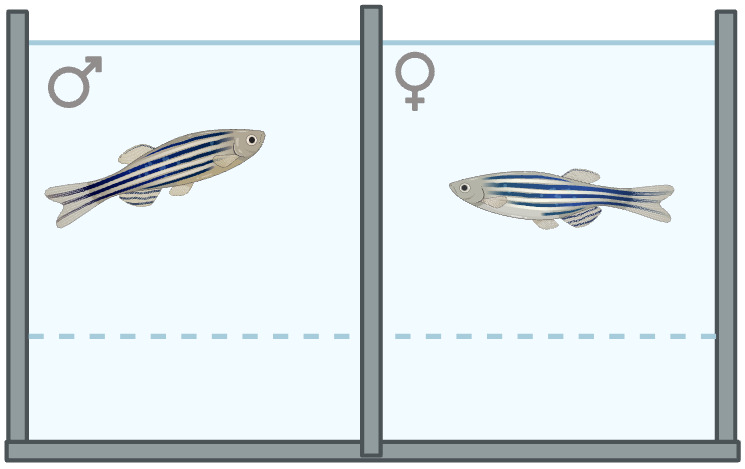
Male and female zebrafish separated by mating divider in a breeding tank

**Figure 2.**
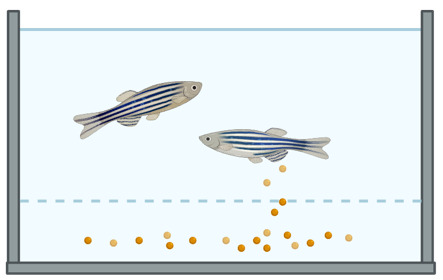
Zebrafish embryos are observed below the breeding mesh

**Figure 3.**
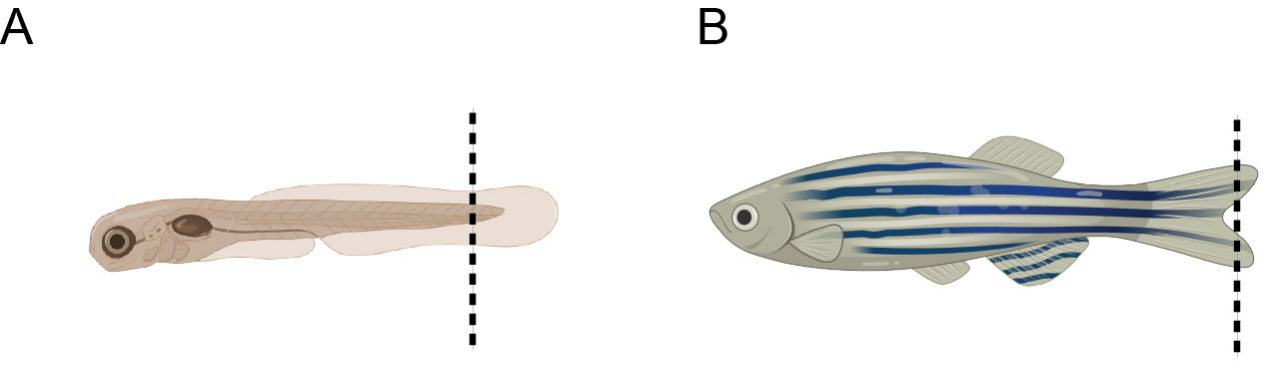
Schematic representation of the recommended tail fin clipping site, indicated by the dashed line. (A) Embryo. (B) Adult fish.

1. BioRender ( https://biorender.com ). The following figures were created using BioRender: Figure 1, https://BioRender.com/q63b896; Figure 2, https://BioRender.com/s08e605; Figure 3, https://BioRender.com/p60f468

## Procedure

**A. Setting up a zebrafish cross**

1. Prepare clean breeding tanks by filling them with system water.

2. Select sexually mature and healthy zebrafish pairs (heterozygous mutants).

3. In the evening, introduce the chosen male and female zebrafish to the breeding tank, separated by the mating divider (Figure 1).

4. Place the breeding setup in a breeding room under a 14/10 h light/dark cycle.

5. The next morning, remove the divider when the lights are turned on.

6. Monitor mating behavior and inspect for eggs.

**B. Collecting embryos**

1. Once mating has occurred and eggs are found at the bottom of the tank (Figure 2), remove the adult zebrafish from the tank using a net and transfer them to a new tank.

2. Collect eggs from the breeding tank into a strainer. Rinse the embryos by gently pouring E3 medium (see Recipes) over the strainer using a squeeze bottle until debris (or feces) is removed.

3. Transfer the embryos from the strainer into a 10 cm Petri dish filled with 15 mL of E3 medium.

4. Incubate the embryos at 28.5 °C with a 14/10 h light/dark cycle for the duration required for the experimental design.

*Note: After each use, clean and disinfect the breeding tanks and nets to maintain fish health.*

**C. Embryo care and maintenance**

1. Regularly monitor embryo development and remove any unfertilized or unhealthy embryos.

2. After gastrulation, which occurs approximately 10 h post-fertilization, add 5 mL of 10× PTU stock solution (see Recipes) to 50 mL of E3 medium (final concentration: 0.003%) to prevent pigment formation.

*Note: PTU is not required if the phenotype of the genetic mutant is visible regardless of pigmentation or if the zebrafish is genetically modified to not develop pigment.*

3. Maintain healthy embryos and collect them (see section D2) at the required timepoint for genotyping.

*Note: The timepoint for embryo collection should be adjusted based on the requirements of the respective experiment.*

4. (Optional) The embryos can be stored in 4% PFA at 4 °C overnight, replaced with 1% PFA the next morning, and stored at 4 °C for up to one week.

**D. Genomic DNA isolation**

1. Prepare the embryo lysis buffer in PCR tubes (see Recipes). Depending on the number of embryos to genotype, you could use individual, 8-strip tubes, or 96-well PCR plates.

2. Collect tissue samples. There are three ways to collect tissues; the most appropriate one should be selected based on your requirements.

a. For live embryos, dechorionate them at the required developmental stage using forceps, anesthetize the embryos with tricaine solution (final concentration 0.02%), cut, and transfer the embryo tails (Figure 3A) to 50 μL of embryo lysis buffer. This is a non-survival procedure on embryos.

b. For adult fish, anesthetize with tricaine 1× solution (final concentration 0.016%), perform a tail fin clip (Figure 3B), and transfer it to 50 μL of embryo lysis buffer. Care should be taken to avoid cutting beyond what is shown in the image, as doing so may cause harm or increase the risk of morbidity in the fish.

c. For 4% PFA fixed embryos, perform the following steps the next day:

i. Cut the embryo tails and transfer them to 1× PBS (see Recipes).

ii. Wash with 1× PBS three times for 5 min each.

iii. Transfer the tissue to 50 μL of lysis buffer.

**Caution:** Decontaminate forceps using 70% ethanol between handling each embryo to avoid cross-contamination of genomic DNA.

3. After immersing the tissue in 50 μL of lysis buffer, incubate the PCR tube containing tissues at 98 °C for 10 min and at 4 °C for 2 min using a PCR machine.

4. Add 5 μL of proteinase K (10 mg/mL) and incubate at 37 °C overnight.

5. The next day, quench the sample at 98 °C for 10 min and at 4 °C for 5 min using a PCR machine to inactivate proteinase K.

6. Centrifuge samples at 1,500× *g* for 15 min at 4 °C.

7. Collect 40 μL of the supernatant into a fresh PCR tube for PCR amplification or store the genomic DNA at -20 °C for future use.

**E. PCR for genotyping**

1. Design genotyping primers spanning the specific region of interest in a way that the region containing the genetic mutation can be amplified and distinguished by a restriction digestion site.

**Tip:** In case the genetic mutation does not create or eliminate a restriction site, design a long primer around 40 nucleotides, creating a restriction site on the genetic mutation. Upon amplification and restriction digestion, a 40-nucleotide difference in PCR product can be resolved on a 4% gel.

2. Prepare the PCR reaction mix as follows (Table 1):

**Table 1.** PCR reaction mix

PCR reagents	Volume (μL)
PCR master mix (Recipe 3)	19
Forward primer (100 mM)	0.04
Reverse primer (100 mM)	0.04
AmpliTaq Gold^TM^ DNA polymerase	0.015
Genomic DNA (template)	1

*Note: To minimize pipetting errors in PCR reactions, especially since genotyping involves multiple embryos, prepare a master mix containing all components (except template DNA) for multiple reactions at once. This reduces small-volume pipetting, ensuring consistency and minimizing errors.*

3. Run the PCR using the following cycle conditions (Table 2):

**Table 2.** PCR cycle conditions

Steps	Temperature	Duration	
Initial denaturation	96 °C	6 min	
Denaturation	95 °C	30 s	45 cycles
Annealing	* °C	30 s	
Extension	72 °C	30 s	
Final extension	72 °C	7 min	
	4 °C	3 min	
	15 °C	hold	

*Note: Adjust the annealing temperature accordingly for different primer sets.

*Note: While this protocol is optimized for AmpliTaq Gold, it can be adapted for other polymerases. Users should adjust cycling conditions (e.g., initial denaturation step, annealing temperatures, and extension times) and ensure they use the recommended buffer and MgCl_2_ concentration provided with their enzyme of choice. For high-fidelity polymerases, shorter extension times may be sufficient.*

4. After PCR, check for product amplification by resolving 5 μL of the PCR product on a 4% agarose gel for 80 min (150 V).

5. Upon successful product amplification, proceed to restriction enzyme digestion as follows (Table 3):

**Table 3.** Restriction enzyme digestion reaction

Reagents	Volume (μL)
PCR product	10
MboII enzyme	0.1
10x (L) buffer	2
MilliQ H_2_0	3

6. Resolve 5 μL of the enzyme-digested PCR product on a 4% agarose gel for 80 min (150 V). This setting can resolve wild-type, mutant, and heterozygous genotypes.

**Tip:** Agarose gel resolving conditions need to be standardized based on the size differences of the bands for each mutant.

## Validation of protocol

**Electrophoresis of PCR-amplified DNAs in an agarose gel to distinguish genotypes: wild-type, heterozygous, and homozygous mutants**

**Figure 4.**
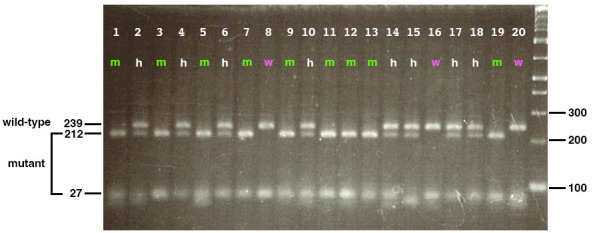
Genotyping of 20 embryos produced by *banp^rw337^* heterozygous parent fishes. When genomic DNA containing a *banp^rw337^* mutation site is amplified with PCR using a specific set of primers, subjected to MboII digestion, which cuts only the mutant-derived genomic DNA, and fractionated by electrophoresis in a 4% agarose gel, three band patterns appear: a single upper band at 239 bp for wild-type embryos (magenta), a single lower band at 212 bp for homozygous mutant embryos (green), and two bands at 212 and 239 bp for heterozygous embryos (white). The right-most lane indicates the DNA ladder maker.

**Figure 5.**
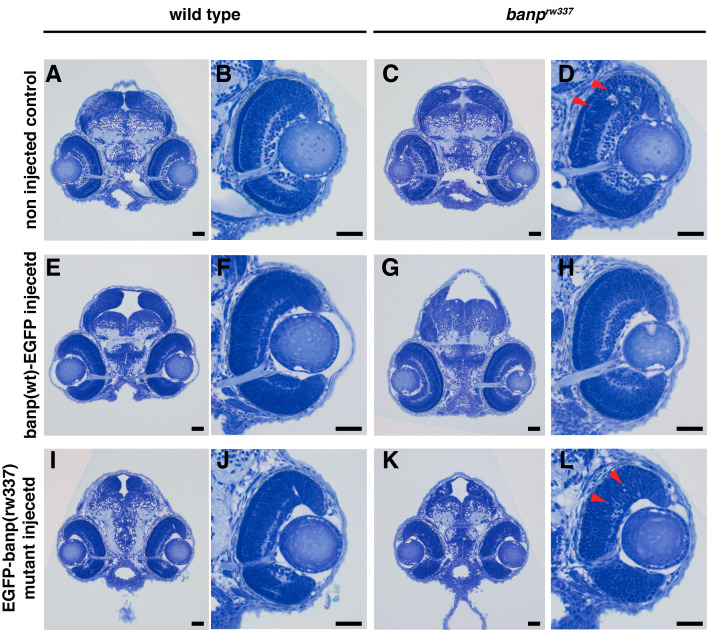
Toluidine blue–stained tissue morphology of *banp^rw337^* wild-type vs. homozygous mutants at 54 hours post fertilization (hpf). (A) Wild-type embryo head. (B) Wild-type retina. (C) *banp^rw337^* mutant embryo head. (D) *banp^rw337^* mutant retina. C and D show pyknotic cells indicating cell death. The red arrow indicates pyknotic cells. Scale bars: 20 μm. Adapted from [5].

To validate the PCR-based genotyping method, genomic DNA from zebrafish embryos (*banp^rw337^*) was amplified using specifically designed primers (forward: 5′-CGATGTTGATATCCATCAGTCAGGCGATC-3′; reverse primer: 5′-GGTGCTGGTGTATAAATCACATGACCTATGGTCCTCTT-3′) and subjected to restriction enzyme digestion using MboII. The resulting PCR products were analyzed by electrophoresis on a 4% agarose gel to distinguish wild-type, heterozygous, and homozygous mutant embryos. This validation step confirms the reliability of the genotyping protocol in differentiating genetic variants based on their banding patterns. As shown in Figure 4, wild-type embryos display a single upper band at 239 bp, homozygous mutants exhibit a single lower band at 212 bp, and heterozygous embryos present both bands at 212 and 239 bp. Furthermore, to confirm the genotypes at the tissue morphology level, toluidine blue staining was performed on sectioned embryos. As illustrated in Figure 5, differences in tissue structure between wild-type and mutant embryos were evident, with mutant embryos displaying pyknotic cells indicative of cell death. These results demonstrate that PCR amplification, restriction digestion, and subsequent histological analysis effectively identify zebrafish genotypes at early developmental stages.

**Plastic sectioning and toluidine blue staining to visualize early phenotypic defects of zebrafish embryos**

*Note: Toluidine blue staining can be used to examine the tissue morphology of wild-type vs. genetic mutant embryos genotyped using the PCR-based method.*

1. Dechorionate embryos using forceps and fix them with 4% PFA at 4 °C overnight.

2. Wash with 1× PBS three times for 5 min each at room temperature.

3. Replace 1× PBS with 100% ethanol in a serial manner (Table 4):

**Table 4.** Ethanol dehydration steps for embryo processing

Ethanol (%)	Time (min)
30%	2
50%	2
70%	2
100%, 2×	2

4. Transfer the embryos to a 1:1 mixture of 100% ethanol and JB-4 solution A for 3 h at room temperature.

5. Immerse the embryos in JB-4 solution A overnight at 4 °C.

6. Embed the embryos in a rectangular silicone mold using JB-4 (A + B) solution mixed in a 25:1 ratio and adjust the embryos to the desired orientation.

7. Cover the mold with transparent cellophane paper to restrict air exposure and allow the solution to solidify.

8. After solidification, trim the sample and mount it on a 2 cm wooden block.

9. Cut the mounted sample into 5–7 μm thin sections using a microtome.

10. Gently arrange each section on top of distilled water domes on a glass slide using forceps and dry on a hot plate.

11. After drying, immerse the slide in 0.1% toluidine blue solution for one dip.

12. Wash the slides with distilled water until excessive toluidine blue dye is removed from the tissues.

13. Dry the slides on a hot plate and mount them with inclusion reagent.

14. Image the stained slides using a microscope.

This protocol or parts of it has been used and validated in the following research articles:

• Babu et al. [4]. Banp regulates DNA damage response and chromosome segregation during the cell cycle in zebrafish retina. *eLife.* 11: e74611.

• Masai et.al. [6]. N-cadherin mediates retinal lamination, maintenance of forebrain compartments and patterning of retinal neurites. *Development* 130(11): 2479–2494.

• Yamaguchi et.al. [7]. Histone deacetylase 1 regulates retinal neurogenesis in zebrafish by suppressing Wnt and Notch signaling pathways. *Development* 132(13): 3027–3043.

• Yamaguchi et al. [8]. Mutation of DNA primase causes extensive apoptosis of retinal neurons through the activation of DNA damage checkpoint and tumor suppressor p53. *Development* 135(7): 1247–1257.

• Nishiwaki et al. [9]. The BH3-Only SNARE BNip1 Mediates Photoreceptor Apoptosis in Response to Vesicular Fusion Defects. *Dev Cell.* 25(4): 374–387.

• Iribarne et al. [10]. Aipl1 is required for cone photoreceptor function and survival through the stability of Pde6c and Gc3 in zebrafish. *Sci Rep.* 7(1): e1038/srep45962.

## General notes and troubleshooting

**General notes**

The described genotyping and tissue analysis protocol proves to be valuable not only for characterizing mutant phenotypes but also for determining the outcome of rescue experiments aimed at restoring normal development in zebrafish genetic mutants. After conducting a rescue experiment, such as reintroducing the wild-type gene or employing CRISPR/Cas9 gene editing, the resulting embryos can be collected and subjected to the PCR-based genotyping method to identify successfully rescued mutant embryos from the wild type. Subsequently, the genotyped embryos can undergo plastic sectioning and toluidine blue staining, as explained above, enabling visualization and comparison of tissue morphology across wild-type, mutant, and putatively rescued mutant embryos. If the rescue is effective, the tissue architecture in the rescued mutant embryos should closely resemble that of the wild type. Thus, this protocol facilitates the validation of rescue experiments at the tissue level.

Plastic sectioning and toluidine blue staining described in the validation of protocol offer advantages for visualizing early phenotypic defects in zebrafish embryos, such as clearer tissue morphology with thinner sections. This method is particularly useful for examining developmental defects in wild-type vs. genetic mutant embryos, especially when combined with PCR-based genotyping. It is cost-effective, simple, fast, and accessible for routine use in most labs. Alternatives include cryosectioning, which preserves tissue integrity but requires specialized equipment, and paraffin sectioning, which is more time-consuming but allows for a wide range of staining options. These alternative methods also allow for immunofluorescence staining, enabling the use of antibodies to detect specific proteins or markers in tissues. The choice of the method should be based on the specific research question and whether additional staining techniques like immunohistochemistry or immunofluorescence are needed.

**Troubleshooting**

Problem 1: PCR gel shows no bands.

Possible causes: a) PCR cycle conditions were not optimized; b) primer was not optimized; c) genomic DNA in the reaction is too low or too high.

Solutions: a) Optimize PCR cycle conditions for primer [11]; b) optimize primers [11]; c) check the concentration of genomic DNA extracted from zebrafish. Too much or too little DNA can be problematic.
